# Population Viability and Conservation Strategies for the Eurasian Black Vulture (*Aegypius monachus*) in Southeast Europe

**DOI:** 10.3390/ani11010124

**Published:** 2021-01-08

**Authors:** Kyriakos G. Dimitriou, Evangelos G. Kotsonas, Dimitrios E. Bakaloudis, Christos G. Vlachos, Graham J. Holloway, Reuven Yosef

**Affiliations:** 1 Lab. of Wildlife and Freshwater Fish, School of Forestry and Natural Environment, Aristotle University of Thessaloniki, P.O. Box 241, 54 124 Thessaloniki, Greece; kyriakdg@hotmail.com (K.G.D.); kotsonas@for.auth.gr (E.G.K.); cvlachos@for.auth.gr (C.G.V.); 2 School of Biological Sciences, University of Reading, Whiteknights, Reading, Berkshire RG6 2AS, UK; g.j.holloway@reading.ac.uk; 3 Ben Gurion University of the Negev—Eilat Campus, P.O. Box 272, Eilat 88000, Israel; ryosef60@gmail.com

**Keywords:** *Aegypius monachus*, population viability analysis, supplementary feeding site, poison, wind farm, reintroduction, cinereous vulture

## Abstract

**Simple Summary:**

Vultures have suffered dramatic declines worldwide. Using population viability models for the Eurasians Black Vulture population in Southeast Europe, we found that the current population is viable for a period of 100 years. However, high levels of poisoning, food reduction, and increase of wind farms make the population prone to extinction. With no supplementary feeding, the population showed an increased but low extinction risk. Our results suggest that removing and translocating juveniles from the extant population to a historic breeding area would not negatively affect the existing population. We suggest the establishment of more supplementary feeding sites and the reintroduction of the species to former breeding areas, along with the elimination of threats posed by poisoning and wind farms in order to increase the population and enhance the dispersal of the species across its historic range in Southeast Europe.

**Abstract:**

The Eurasian Black Vulture is a globally threatened raptor that in Southeast Europe only occurs in an isolated population in Greece. We examined the population viability for the species under demographic fluctuations and conservation scenarios. The current population showed no possibility of extinction for the next 100 years. However, simulated scenarios showed that the most important factor affecting the viability of the species was medium and high poisoning, leading to 94.8% and 100% probability of extinction, respectively. Furthermore, high reduction of supplementary feeding highlighted an 18.6% extinction possibility. Also, a high increase of wind farms in the area may result in 17.4% extinction possibility. Additionally, the non-establishment of the feeding station in 1987 in the study area would have resulted in an extinction risk of 7%. The species can be translocated to the Olympus National Park by releasing 80 juveniles over 10 years. The implementation of the conservation scenarios concerning the establishment of a supplementary feeding site network, and the reintroduction of the Eurasian Black Vulture in its historic range, along with the elimination of threats posed by poisoning, low food availability, and wind farms would increase the probability of the species persistence and allow the population to become a source for dispersal across Southeast Europe.

## 1. Introduction

In recent years, the species extinction rate has accelerated worldwide and is a thousand times higher than through natural processes alone [1]. Small, isolated populations are especially vulnerable to extinction [2] due to deterministic and stochastic threats [3]. The conservation of such populations is challenging [4].

Vultures are considered the most threatened raptors worldwide [5,6]. A variety of factors, including contamination with veterinary pharmaceuticals [7,8], poisoning [9,10,11], habitat destruction [12], and low food availability [12,13,14], combined with regulations and enactments [15], human persecution [12], collisions with wind turbines [16], electrocution and collisions with power lines [17], and anthropogenic disturbance [18], have contributed to the decline of vultures worldwide. In Europe, vulture population declines began in the mid-19th century leading to local extinctions of some species [6]. In the early 21st century, vulture populations showed a more stable and slightly increasing trend due to changes in European legislation [13] and intensive management and conservation [13,19]. Establishment of supplementary feeding sites (SFS), reintroduction, monitoring, and public education programs [20] have produced encouraging results. Establishment of SFS has proved to be a powerful management tool for the conservation of vultures and other carrion-eating birds [21,22,23,24,25], and has helped to eliminate threats posed by veterinary drugs [26], poison baits [6,27], and carcass disposal management as a result of sanitary legislation (Regulation CE 1774⁄ 2002) [14,19,22,28]. Moreover, a very promising conservation strategy has been the successful reintroduction of different vulture species into parts of their European historic ranges. Bearded Vultures (*Gypaetus barbatus*) were reintroduced in the European Alps [29] and Spain, Griffon Vultures (*Gyps fulvus*) were released in France [30], while Eurasian Black Vultures (*Aegypius monachus*) were reintroduced in France and parts of the Iberian Peninsula [31]. From an ecosystem service perspective, an additional incentive is the cultural service provided by vultures (i.e., ecotourism) [32].

**Figure 1:**
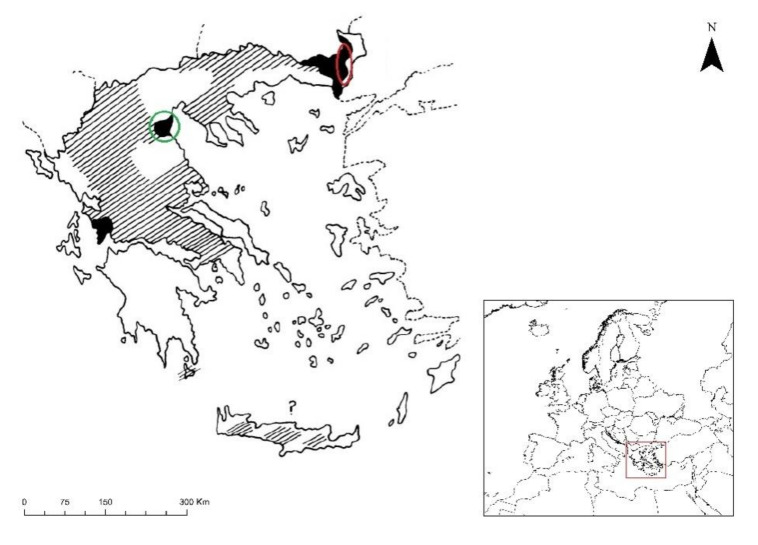
Historic and current distribution of the Eurasian Black Vulture (*Aegypius monachus*) in Greece, Southeast Europe (red square). Hatched areas show the distribution of the species in 1850, while solid black areas show the distribution in 1980. The red circle shows the current distribution of the species in the DLS-NP, while the green circle shows the Olympus National Park. The map is modified by [39].

The Eurasian Black Vulture is nearly the largest raptor in the world requiring large suitable breeding areas [33]. It is a solitary species nesting on top of mature trees. The species is globally classified as ‘Near Threatened’ [34] and ‘Endangered’ in Greece [35]. During the 20th century, the species disappeared from several European countries [34,36] and nowadays shows a patchy distribution across the continent. In Southeast Europe, the species breeds only in Greece in the Dadia-Lefkimi-Soufli Forest National Park (hereafter DLS-NP) [21,35,37]. Once a common species throughout continental Greece, Eurasian Black Vulture suffered a dramatic decline after 1950 [38] (Figure 1). Except from Thrace, the species was common in Thessaly and especially on mounts Olympus and Othrys. In 1984, the species numbered 24 individuals (10 breeding pairs) in the area of the DLS-NP [21]. During the same period, the Olympus population consisted of 7 individuals (2 breeding pairs), that went extinct during the 1990s. In 1987 a supplementary feeding site (SFS) was created in the DLS-NP in order to reinforce the species population, which in 1997 had increased to 59 individuals (21 breeding pairs) [21]. However, until 2005, the population did not significantly increase, probably due to the illegal use of poisoned baits in the area [37]. During the last decade, the population has increased further, and in 2020, there were 120–130 individuals (35 pairs) in the area [31].

The aim of our study was to assess the probability of persistence of the isolated Eurasian Black Vulture population in Southeast Europe and to investigate the factors affecting the population, specifically poisoning, reduction of supplementary feeding, and increase of wind farms. Furthermore, we aimed to evaluate the effectiveness of two conservation scenarios: (a) the population trend under non-establishment of the SFS in 1987, and (b) the viability of a reintroduced population to Olympus National Park in order to improve the species conservation status in the region and to influence the current management, protection, and conservation of the species in Southeast Europe.

## 2. Materials and Methods

### 2.1. Data Collection and Model Parameter Input

Population viability analysis (PVA) is a tool for endangered species management and conservation due to the focus on species limitation factors [40]. For this reason, vultures constitute popular target species [41,42]. We used the Vortex simulation software (version 10.3.8) in order to build a PVA model [43] with the demographic parameters of the Greek population of Eurasian Black Vulture (Table 1). The data were collected from published studies [21,37] and researchers who have worked on the ecology and protection of the species (unpublished data). The simulations were started from the year 2005, due to lack of available data for the subsequent years, and when the initial population size was 75 individuals. We calculated the stochastic population growth rate (r_stoch_) by dividing the population size of a given year to the size of the population of the previous year for each scenario. We performed 500 iterations in order to estimate the likelihood of the future existence of the species for a period of 100 years. The deterministic growth rate (r_det_) was calculated by Vortex.

The population of Eurasian Black Vulture in Greece is disjunct and isolated; hence we assumed that immigration and emigration are unlikely to occur. Individual vultures have been recorded moving locally to Bulgaria, but none to the closest breeding population in Western Turkey which is ca. 400 km away (personal observations). The probability of extinction of the population was obtained by dividing the number of simulations in which the population had disappeared by the total number of simulations. We defined that extinction occurs only when one sex remained in the population. We considered the age of sexual maturity and first breeding [36], and the maximum age of reproduction [44] (Table 1). The sex ratio is 1:1 [45]. Data on age class distribution were not available so the initial population of Eurasian Black Vulture was distributed to the different age classes by the Vortex software according to the expected age distribution calculated from the birth and death rates. The reproductive system was modeled as long-term monogamy [46].

Annually, the Eurasian Black Vulture has one brood and lays only one egg [36]. Reproduction was set as the time the egg was laid, and time of hatching as the starting point of the life cycle. The reproduction of the species was included as density-dependent using data from 1988–2005 [21,37] so that different proportions of adult females bred in different population sizes. The density-dependent reproduction affects the reproductive outcome of raptors and especially vultures [47]. Fecundity was defined as the mean number of nestlings per pair and estimated by the number of fledglings to the total number of territorial pairs [5]. The percentage of breeding females was calculated by the equation provided by VORTEX [48]:

P(N) = [(P(0) − P(K))(N/K)^B][N/(N + A)](1)

where P(0) is the percentage of adult females breeding at low density; P(K) is the percentage of adult females breeding at carrying capacity; N is the initial population size; K is the carrying capacity; B is the steepness parameter, which determines the shape of the curve relating the percentage of adult females breeding to population size; and A is the Allee parameter, which accounts for the decrease in the proportion of females breeding at low densities. The percentage of breeding females at low density were obtained by the productivity values (N of fledglings/N of occupied nests) and the proportion of breeding females at carrying capacity by dividing the number of breeding females by the total number of females (Table 1). Steepness parameter (B) was estimated according to the shape of the curve, and the Allee effect was not considered (Table 1). The environmental variation in reproduction was modeled by entering a standard deviation (SD) for the percent females producing offspring and calculated to 18%. The carrying capacity (K) of the area was estimated by multiplying the maximum number of individuals observed (2015: 135 individuals) by 1.15 and rounding it up to the closest 100 [42] (Table 1). Also, the significantly reduced percentage of the breeding females in this population size [21,37] indicates that the population reaches the carrying capacity of our simulations. The standard deviation of carrying capacity due to environmental variability was estimated at 20 individuals.

The number of broods per year was calculated by dividing the number of incubating pairs by the total number of pairs using data for 1994–2005 [37]. The likelihood of a pair laying one brood per year was 83.44%, while not laying the same year was 16.56%. The distribution of the existence of one offspring per female per brood was calculated automatically by the program as 100%. Also, the percentage of males in the breeding pool was 100%. The loss of heterozygosity was introduced to the models by using the 6.29 default lethal equivalent per diploid chromosome given in Vortex for the initial population. This determines the value at which survival is reduced because of inbreeding [48].

Mortality rates per age class were taken from the literature [49,50] (Table 1) and considered the same for both sexes. Furthermore, we used Vortex’s default values for the standard deviation in mortality due to environmental variability (10% for juvenile mortality; 3% for immature and adult mortalities). We considered these values as we assumed that in the relatively stable environment of the National Park we would not see large annual fluctuations in mortality rates.

### 2.2. Sensitivity Analysis

Sensitivity analysis was used to elucidate the effects of environmental parameters on the viability of the population over the next 100 years. Five alternative scenarios were created (Table 2) and the levels in each scenario selected appropriately. The stochastic growth rates of each scenario were compared to the stochastic rates of the basic scenario. We modeled the effects of decrease in the supplementary feeding (increase mortality and decrease fecundity) and the development of wind farms (increase mortality). The intensity levels were chosen in a similar way as provided by García-Ripollés and López-López [41] for PVA on Griffon and Egyptian Vultures. Also, poisoning was modeled as a catastrophic event with three intensity levels [42]. Catastrophic events are unpredictable, and the frequency of occurrence was set at 20% probability of occurring in any one year. Each event was modeled under a severity factor (range: 0–1) [48]. For example, an effect of 0.90 indicates 10% decrease in the parameter referred to.

The low poisoning scenario was chosen in order to estimate the minimum viable population (MVP) of Eurasian Black Vulture in Greece and Southeast Europe. This is important because although MVP is an appropriate indicator for the protection and conservation of threatened populations, it is not constant and varies between species, and subpopulations of the same species [51]. The MVP was estimated by altering the initial population size and defining the smallest population size with a possibility of extinction of less than 1% for the next 100 years.

### 2.3. Conservation Scenarios

#### 2.3.1. Non-Establishment of the SFS in 1987

We used demographic data from 1984–1987 [21]. The population of the species was low and ranged between 22–36 individuals. These numbers were the maximum for the area, so the carrying capacity was estimated at 100 individuals, by multiplying the maximum number of individuals observed (1987: 36 individuals) by 1.15 and rounding it up to the closest 100 [42]. The standard deviation of carrying capacity due to environmental variability was estimated at 10 individuals. The percentage of breeding females at low density P(0) and at carrying capacity P(K) were estimated as 47.5% and 27.81%, respectively. The environmental variation in reproduction was modeled by entering a standard deviation (SD) for the percentage females producing offspring and calculated as 5%. The simulations started at 1987, and the initial population was set at 36 individuals. All other parameters remained stable as in the basic scenario.

#### 2.3.2. Reintroduction of Eurasian Black Vulture to Olympus National Park

The possible reintroduction of the Eurasian Black Vulture to Olympus National Park was based on two sub-scenarios. First, we removed individuals to implement a translocation, while the percentage survival during translocation was set at 100%. Eight juveniles per year were removed for 10 years, i.e., a total of 80 individuals removed from the DLS-NP population. The removal is planned to occur in 2025–2034. The sex ratio was 1:1 due to the maximization of the growth rate and the success of reintroduction in this monogamous species.

Subsequently, juveniles removed from the DLS-NP population were reintroduced to Olympus National Park. The initial population size was 8 individuals (4 males, 4 females). The carrying capacity of the new population was set at 100 individuals, and the standard deviation due to environmental variability was 10.

### 2.4. Statistical Analyses

We performed the non-parametric Friedman ANOVA to examine statistical differences between the stochastic growth rates of the basic scenario and the levels of each alternative scenario. All statistical analyses were conducted using the statistical package STATISTICA version 10 [52]. The level of significance for all tests was set as 0.05.

## 3. Results

### 3.1. Basic Scenario

The population of Eurasian Black Vulture on the DLS-NP increased during the first years of simulations and remained constant with oscillations around the carrying capacity for a period of 100 years. The mean deterministic growth rate (*r_det_* range_100 years_ = 0.0265) and the stochastic growth rate (*r_stoch_* range_100 years_ = 0.0134) of the population were positive, while the probability of extinction was zero.

### 3.2. Sensitivity Analysis

**Figure 2:**
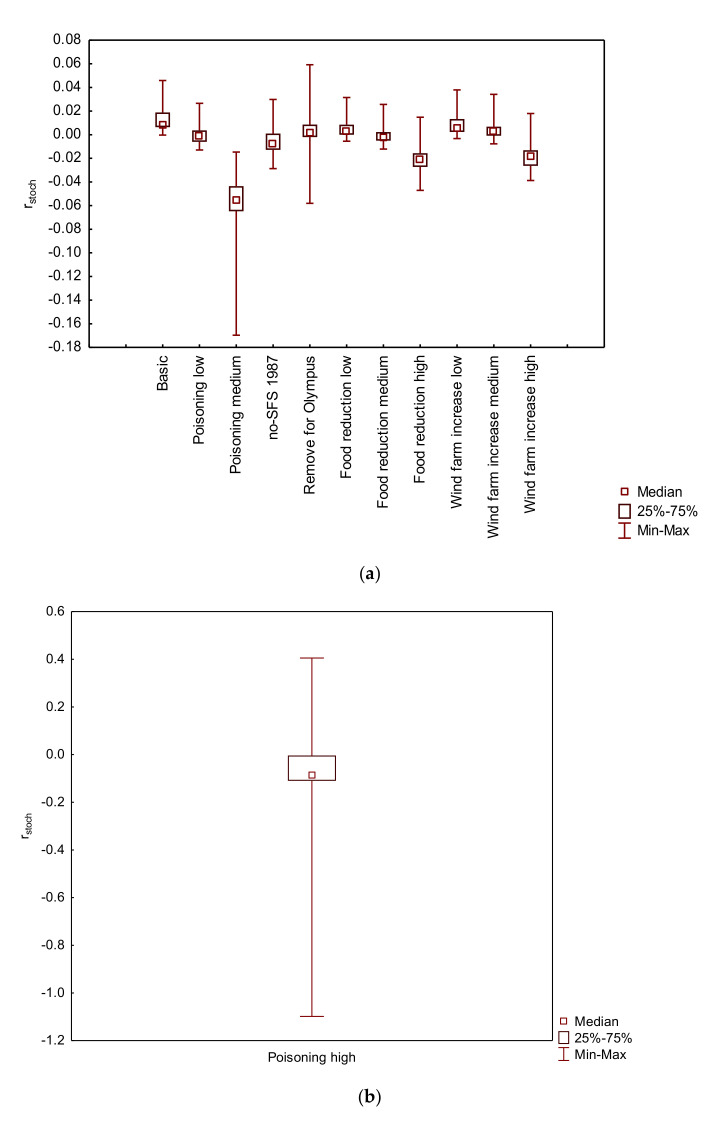
Comparative stochastic population growth rates (r_stoch_) under different scenarios: (**a**) Basic and alternative scenarios and (**b**) High poisoning scenario, simulated (100 years) in a Population Viability Analysis in Southeast Europe for the Eurasian Black Vu lture (*Aegypius monachus*).

Sensitivity analysis showed significant differences in population trends between the basic and each alternative scenario (Figure 2a,b).

**Figure 3:**
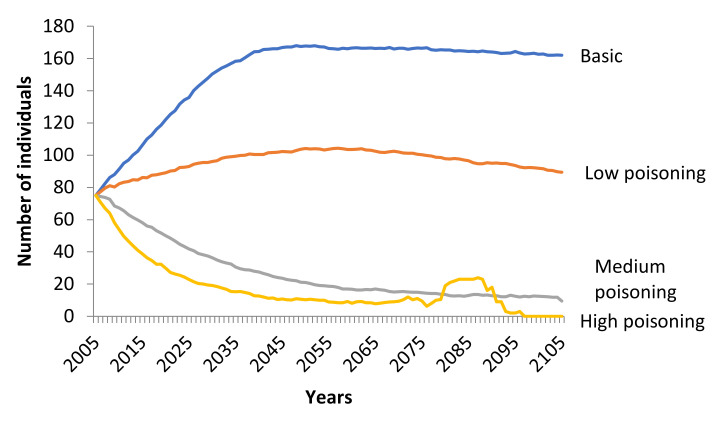
Trend of the Eurasian Black Vulture (*Aegypius monachus*) population, under low, medium, and high poisoning scenarios simulated (100 years) in Southeast Europe. Poisoning inserted as a catastrophic event with frequency of occurrence 20%. The effect on fecundity was set at 0.90 (10% decrease) and the effect on survival was set at 0.90 (10% decrease), 0.70 (30% decrease) and 0.50 (50% decrease) for low, medium, and high poisoning, respectively. All other parameters remained stable as in the basic scenario.

The most important factor affecting the viability of the Eurasian Black Vulture population was poisoning, which was introduced as a catastrophic event. Low poisoning resulted in negative growth rate (*r_stoch_* = −0.0001) (Friedman ANOVA χ^2^_100,3_ = 201.7320, *p* < 0.001), with a low possibility of extinction (PE_100 years_ = 0.0160) and a mean time of extinction (TE) of 90.3 years. On the other hand, medium (*r_stoch_* = −0.0520) and high (*r_stoch_* = −0.1031) poisoning lead to a significant reduction of the population (Friedman ANOVA χ^2^_100,3_ = 201.7320, *p* < 0.001), with the possibility of extinction 94.8% and 100%, respectively, over the next 100 years (Figure 3). Moreover, the mean TE of the population under medium poisoning was 56.2 years, while the mean time of extinction under high poisoning was 29.8 years.

**Figure 4:**
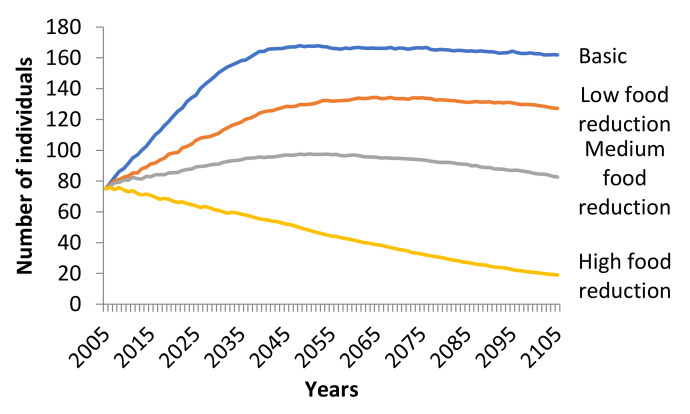
Trend of the Eurasian Black Vulture (*Aegypius monachus*) population, under low, medium and high reduction of supplementary feeding scenarios simulated (100 years) in Southeast Europe. Reduction of supplementary feeding had an effect on fecundity (low: −10%, medium: −10%, high: −20%) and all age mortality (low: +10%, medium: +20%, high: +30%). All other parameters remained stable as in the basic scenario.

Decrease of supplementary feeding affected the growth rate of the Eurasian Black Vulture population (Figure 4). Low food reduction showed a positive growth rate (*r_stoch_* = 0.0058) with no possibility of extinction. Medium and high reduction of supplementary feeding resulted in negative growth rates (medium: *r_stoch_* = −0.0005, high: *r_stoch_* = −0.0202) and differed significantly from the basic scenario (Friedman ANOVA χ^2^_100,3_ = 271.7387, *p* < 0.001). The possibility of extinction of the population was 0.6% for medium and 18.6% for high reduction of supplementary feeding. The mean TE under medium and high supplementary food reduction was 88.3 and 84.1 years, respectively.

**Figure 5:**
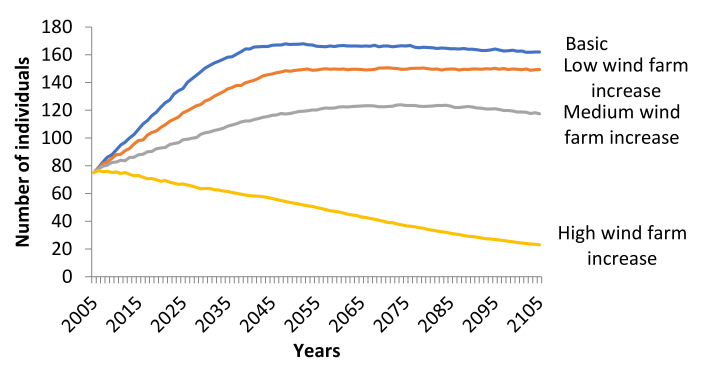
Trend of the Eurasian Black Vulture (*Aegypius monachus*) population under low, medium and high increase of wind farms scenarios simulated (100 years) in Southeast Europe. Increase of wind farms had an effect on all age mortality (low: +10%, medium: +20%, high: +40%). All other parameters remained stable as in the basic scenario.

High increase of wind farms resulted in negative growth rate (*r_stoch_* = −0.0178) and differed significantly from the basic scenario (Friedman ANOVA: χ^2^_100,3_ = 230.1532, *p* < 0.001) (Figure 5). The possibility of extinction of the population was 17.4% and the mean TE was at 86.5 years.

Moreover, the MVP of the Eurasian Black Vulture in DLS-NP, estimated at 50 individuals, ensured 99% survival of the population over the next 100 years. This number was estimated in spite of the effect of low poisoning, in order to evaluate the trend of the population under catastrophic events, with direct consequences for the population.

### 3.3. Conservation Scenarios

#### 3.3.1. Non-Establishment of the SFS in 1987

**Figure 6:**
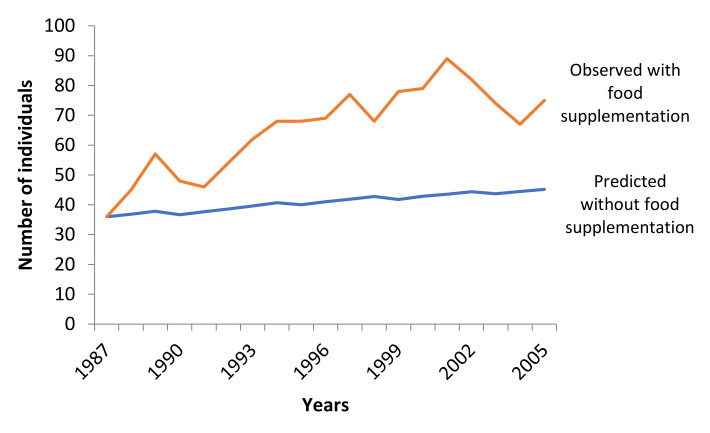
Observed population trend of the Eurasian Black Vulture (*Aegypius monachus*), under supplementary feeding in the Dadia-Lefkimi-Soufli Forest National Park [21,37], and the predictive trend of the population under the scenario of non-establishment of the supplementary feeding site, during the years 1987–2005. The initial population size was 36 individuals; the carrying capacity (K) was set at 100 ± 10 standard deviation in K due to environmental variability. The percentages of breeding females at low density P(0) and at carrying capacity P(K) were estimated as 47.5% and 27.81%, respectively, and the environmental variation in reproduction was calculated to 5%. All other parameters remained stable as in the basic scenario.

The simulations demonstrated that without the establishment of the SFS in 1987 the trend of the Eurasian Black Vulture population in the DLS-NP would be negative and maintained at low levels (Figure 6). The deterministic growth rate was positive (*r_det_* range_100 years_ = 0.0164), while the stochastic growth rate was negative (*r_stoch_* range_100 years_ = −0.0031) and the possibility of extinction of the species over the next 100 years was relatively low at 7%. Moreover, the mean TE was 81.2 years.

#### 3.3.2. Reintroduction of Eurasian Black Vulture to Olympus National Park

**Figure 7:**
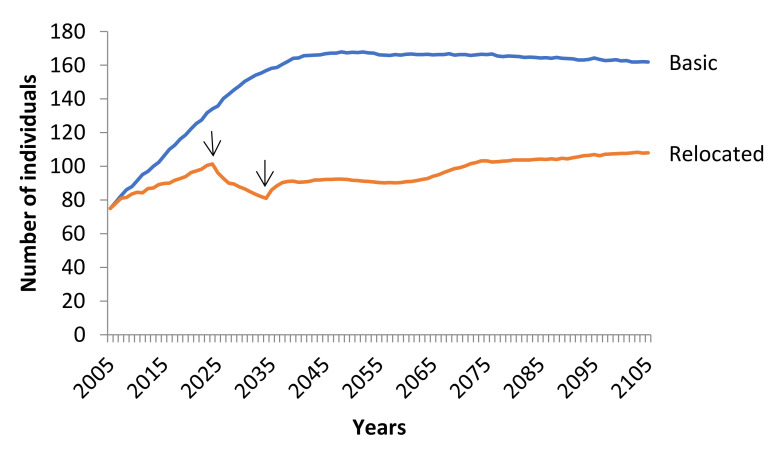
Trend of the population of the Eurasian Black Vulture (*Aegypius monachus*) under the scenario of removing individuals from the Dadia-Lefkimi-Soufli Forest National Park population in order to reintroduce them in the Olympus National Park. Eight juveniles per year removed for consecutive 10 years, i.e., a total of 80 individuals removed from the population of Dadia-Lefkimi-Soufli Forest National Park. The removal was planned to occur during the years 2025–2034 indicated by the arrows. All other parameters remained stable as in the basic scenario.

The removal of eight juvenile vultures per year for 10 continuous years from the DLS-NP showed no effect on the viability of the existing population and the possibility of extinction after removal was zero. The mean stochastic growth rate of this scenario (*r_stoch_* = 0.0028) differed significantly from the stochastic growth rate of the basic scenario (Friedman ANOVA χ^2^_100,1_ = 37.5858, *p* < 0.001) (Figure 7). However, the population showed an extremely low probability of extinction (PE_100 years_ = 0.0080) and a mean time of extinction (TE) at 92.5 years. The new reintroduced population to Olympus showed a positive trend (*r_stoch_* = 0.0275) and the possibility of extinction was zero.

## 4. Discussion

Our results from the basic scenario indicate that the population of the Eurasian Black Vulture is viable for the next 100 years under the current conservation strategy. However, alternate scenarios suggest that extinction could occur. Different levels of poisoning showed a progressive possibility of extinction. Also, medium and high reduction of supplementary feeding as well as the establishment of a high number of wind farms negatively affected the population growth and predicted the extinction of the population. Moreover, the establishment of the SFS in 1987 positively affected the population as observed by the positive trend, while the absence of the SFS predicted a moderate population increase and a low possibility of extinction. Finally, 80 juvenile vultures could be removed in a period of 10 years (8 individuals per year) from the current population in order to form a new viable reintroduced population in the Olympus National Park.

Poisoning is considered to be one of the greatest threats to vultures in Europe [9,11,28,31,53,54]. Our results showed that poisoning is the most crucial factor negatively affecting the population of Eurasian Black Vulture in the area of the DLS-NP resulting in high extinction probabilities under medium (94.8% possibility of extinction) and high (100% possibility of extinction) poisoning levels. In southeast Europe, massive poisoning events resulted in many vulture deaths [53,55]. Our results suggest the absence of control may lead to extirpation of the species from the area. The extensive use of illegal poison baits for the eradication of carnivores (including wolves and dogs) is the main cause of vulture poisoning in the Balkans [53,55]. The reappearance of wolves during the last decade resulted in the occurrence of carcasses accessible to vultures, which in turn were used as poison baits by different social groups [56], resulting in the death of the scavenger species [53,55]. Adult mortality is a crucial factor affecting necrophagous birds and the sustainability of their populations [57]. Adults suffer greater mortality due to their dominance over a carcass and their feeding priority. Adverse effects of poisoning could be reduced by the inclusion of more feeding stations for vultures.

Supplementary feeding is considered a safe food source [6,27]. However, despite the intensive use of the SFS of the DLS-NP, Eurasian Black Vultures show high mobility and continue to exploit wide areas outside the National Park for foraging (pers. obs.). Eurasian Black Vultures from Greece have been observed travelling to Bulgaria in search of food. Outside Greek borders, traditional stock rearing systems exist and favor the presence of different necrophagous species, such as Griffon and Egyptian Vultures [58]. On the other hand, poisoning events outside the Greek borders are quite common and the exposure of vultures to poisoned baits is relatively high [53]. The establishment of an SFS network through Thrace and Bulgaria would facilitate the safer dispersal of the species and help to eliminate poisoning events [53]. The use of a feeding station by adult Eurasian Black Vultures is affected positively by the presence of nearby feeding stations [24]. So, the operation of the second SFS in the area of the DLS-NP, and the establishment of new SFS would result in higher food supplies safe from poison and consequently increase the carrying capacity of the area. As a result, the species could colonize adjacent regions owing to the availability of suitable nesting grounds [59].

Food supplies and nest sites determine carrying capacity for birds of prey in most environments. Therefore, the breeding numbers of a species can be limited if one of these factors is in short supply [5]. Our results suggest that food availability is a crucial limiting factor affecting the population of Eurasian Black Vulture in the DLS-NP [21] while nest sites are abundant. Our results demonstrate that if the feeding station was not created in the study area in 1987, the population of the Eurasian Black Vulture would have declined and exhibit an extinction risk of 7% during the next 100 years. The establishment of the SFS was crucial for the survival of the species in Greece due to the low numbers of livestock during that period. Furthermore, if the species had not been supported through supplementary feeding, the European sanitary policy would have had a devastating effect on the population through the elimination of available food supplies [13,15,19]. Hence, the species would probably be more scarce or extinct from the country.

Vulture restaurants are under debate worldwide due to the positive and negative effects of supplementary feeding [10,15,47]. In the case of Eurasian Black Vulture in Greece, the supplementary feeding had a positive effect on the conservation of the species [21]. After the establishment of the SFS in 1987, the population has increased from 20 to ca. 130 individuals in 2020, while the extinction risk decreased significantly because of the increased fecundity and decreased mortality. Also, the population could further increase and thus buffer against stochastic events, and maybe even become a source for surrounding regions/countries. Sensitivity analysis showed that a high reduction of supplementary feeding could negatively affect the population and highlights an 18.6% probability of extinction. Supplementary feeding reinforces natural food availability [10] and increases pre-adult survival, thus constitutes an important management practice for the conservation of vultures in Europe [25]. Negative effects on necrophagous birds arising by new carcass management can be mitigated through supplementary feeding [14,19], while carcass deposition for vultures in protected and fenced locations is authorized by European legislation [60].

Wind farms pose a great risk for birds of prey and especially vultures [16,61]. An increase of 10–20% in annual mortality per age class showed no effect on the trend of the population of the Eurasian Black Vulture in Greece. However, an increase in mortality of about 40% gave a medium risk of extinction (17.4%) over the next 100 years. Under the current situation, the vulture population has a positive trend and shows no possibility of extinction. Taking into account that Eastern Thrace has been characterized as a Wind Farm Priority Area, a future increase in the number of wind turbines in the area may lead to increased bird strikes and, consequently, to higher mortality rates. According to that scenario, the population of the Eurasian Black Vulture will have difficulty recovering and its extinction may be accelerated. In order to avoid such increase in mortality rates caused by the operation of wind turbines, a spatial management plan should be applied. Across Europe, guidelines for the establishment of wind farms outside wildlife-sensitive areas have been applied [16,62]. Furthermore, at a regional scale, it is proposed to develop species specific sensitivity maps for the efficient placement of wind turbines [62].

During the 21st century, the reintroduction of a species in areas where they formerly existed is a useful management tool in conservation biology [63]. The success of a reintroduction program depends on the number and age of individuals released [49], the elimination of threats [29], and the systematic monitoring of the released individuals [64]. The Eurasian Black Vulture is a species that has been successfully reintroduced in parts of its historic European range [31]. In accordance with our reintroduction scenario, a total of 80 juvenile Eurasian Black Vultures will constitute the basis of a new population in the Olympus National Park. The selection of juveniles constitutes good practice for the reintroduction of vultures as has been demonstrated in the case of Eurasian Black Vulture in France [49] and Bearded Vulture in the Alps [41]. However, some recommend the reintroduction of only adults, like the case of Griffon Vulture in southern France [65]. Juvenile vultures were selected because no significant differences occurred in the stochastic growth rate of the initial population before and after their removal. Olympus National Park constitutes a former breeding area of the species providing a good supply of traditional nest sites. Lack of animal husbandry in the area of the National Park will keep the population safe from illegally poisoned baits that are used by farmers to eradicate large carnivores [54]. Furthermore, food availability is appropriate in the area due to the presence of wild ungulates such as the Balkan Chamois (*Rupicapra rupicapra*), Roe Deer (*Capreolus capreolus*), and Wild Boar (*Sus scrofa*). Additionally, supplementary feeding is a key factor to support the newly introduced individuals [29]. Hence, the establishment of a feeding station will ensure the success of the reintroduction of the new Eurasian Black Vulture population and also help with monitoring. The monitoring of the new population is essential for assessing the success of reintroduction [49].

## 5. Conclusions

This study illustrates the primary conservation actions required to sustain a viable population of Eurasian Black Vultures in Southeast Europe. The maintenance of supplementary feeding by the operation of all SFS in DLS-NP, as well as the restriction of poisoned baits and the efficient placement of wind farms, constitute the major conservation actions recommended. Additionally, the establishment of further SFS in Thrace and Bulgaria, and the reintroduction of the species in its historic range, should be undertaken in order to encourage the dispersal and recolonization of the species throughout its historical range in Southeast Europe.

## Figures and Tables

**Table 1:** Input parameters used in the basic scenario for the Population Viability Analysis of the Eurasian Black Vulture (*Aegypiusmonachus*) in Southeast Europe.

Parameter	Eurasian Black Vulture
Reproductive system	Long-term monogamy
Age of first offspring	6
Maximum age of reproduction	30
Maximum broods per year	1
Sex ratio at birth	1:1
Density-dependent reproduction	Yes
% Breeding females at low density, P(0)	66.9
% Breeding females at carrying capacity, P(K)	30.49
Allee parameter, A	0
Steepness parameter, B	4
% Males in the breeding pool	100
Carrying capacity (K)	200
Initial population size (N)	75
Mortality (%) from age 0 to 1 (SD)	8 (10)
Mortality (%) from age 1 to 2 (SD)	30 (3)
Mortality (%) from age 2 to 3 (SD)	15 (3)
Mortality (%) from age 3 to 4 (SD)	15 (3)
Mortality (%) from age 4 to 5 (SD)	15 (3)
Mortality (%) from age 5 to 6 (SD)	15 (3)
Mortality (%) after age 6 (SD)	2 (3)

**Table 2:** Simulated scenarios used in Population Viability Analysis of the Eurasian Black Vulture (*Aegypius monachus*) in Southeast Europe. Parameters of the Basic Scenario (B) are presented in Table 1. The intensity levels for each alternative scenario were chosen in a similar way as provided by García-Ripollés and López-López [42] and presented the magnitude of change of the demographic parameters in relation to the Basic scenario. Poisoning inserted as a catastrophic event with frequency of occurrence 20% probability to occur in a year. The effect on fecundity was set at 0.90 (10% decrease) and the effect on survival was set at 0.90 (10% decrease), 0.70 (30% decrease) and 0.50 (50% decrease) for low, medium, and high poisoning, respectively.

Scenario	Levels	Fecundity	% Mortality			Catastrophic Events			
			Juvenile (0–1)	Immature (1–6)	Adult >6	Occurrence	Frequency	Effect on Fecundity	Effect on Survival
Basic scenario (Β)		Β	B	B	B	No	-	-	-
Reduction of supplementary feeding	low	−10%	+10%	+10%	+10%	No	-	-	-
	medium	−10%	+20%	+20%	+20%	No	-	-	-
	high	−20%	+30%	+30%	+30%	No	-	-	-
Wind farm increase	low	B	+10%	+10%	+10%	No	-	-	-
	medium	B	+20%	+20%	+20%	No	-	-	-
	high	B	+40%	+40%	+40%	No	-	-	-
Poisoning	low	Β	Β	Β	Β	Yes	20%	0.90	0.90
	medium	Β	Β	Β	Β	Yes	20%	0.90	0.70
	high	Β	Β	Β	Β	Yes	20%	0.90	0.50

## Data Availability

The data presented in this study are available on request from the corresponding author.
